# Relative Quantitation of EFNA1 Expression in Mouse Heart Tissue Histologic Sections Using MALDI-MSI

**DOI:** 10.3390/ijms26041398

**Published:** 2025-02-07

**Authors:** Maria Torres, Laura Gruer, Smrithi Valsaraj, Shaun Reece, Jeremy Prokop, Tonya Zeczycki, Cameron Taylor, Taylor Byers, William Cruz, Kim Kew, Lisandra de Castro Braz, Jitka Virag

**Affiliations:** 1 East Carolina Diabetes and Obesity Institute, East Carolina University, Greenville, NC 27834, USA; 2 Department of Physiology, Brody School of Medicine, East Carolina University, Greenville, NC 27834, USA; 3 Department of Pharmacology and Toxicology, Michigan State University, East Lansing, MI 48824, USA; 4 Department of Biochemistry and Molecular Biology, Brody School of Medicine, East Carolina University, Greenville, NC 27834, USA; 5 Department of Chemistry, East Carolina University, Greenville, NC 27834, USA

**Keywords:** myocardial infarction, MALDI-MSI, EFNA1

## Abstract

EFNA1 (ephrinA1), a highly expressed tyrosine kinase receptor-ligand in healthy cardiomyocytes, is reduced following myocardial infarction (MI). A single intramyocardial injection of chimeric EFNA1-Fc at the time of ischemia mitigates the injury in both reperfused and non-reperfused mouse myocardium by reducing apoptosis, necrosis, and inflammation. Recently, we have successfully imaged and qualitatively identified endogenous EFNA1 pre- and post-MI using matrix-assisted laser desorption ionization mass spectrometry imaging (MALDI-MSI) coupled with a time-of-flight mass spectrometer (MALDI/TOF MS). Building on our previous work, we are currently focused on understanding and characterizing EFNA1’s role in cardiac tissue by developing an integrated quantitative method to determine endogenous levels of EFNA1 using MALDI-MSI technologies. Herein, we have optimized a method for the relative quantitation of endogenous tryptic EFNA1 peptides detected in the murine heart as compared with routine western blotting. In healthy myocardium, there was approximately 50 ng of endogenous EFNA1 per section of 9.43 mm^3^ tissue, or roughly 12 pg/µg of homogenized tissue. MALDI-MSI thus provides a tool for determining the anatomical distribution and relative quantitation of endogenous EFNA1 in cardiac tissue. Future applications of these tools will allow us to investigate the dynamic changes in EFNA1 expression profile that occur in pathological states such as myocardial infarction and upon therapeutic treatments.

## 1. Introduction

EFNA1, an endogenous tyrosine kinase receptor-ligand expressed in the cellular membrane of healthy cardiomyocytes, is the only ligand in the ephrin (Eph) family known to bind to all of the EphA receptors [1]. During a myocardial infarction (MI) event, EFNA1 expression decreases in injured cardiomyocytes [2]. Previous work from our group has shown that a single intra-myocardial injection of chimeric EFNA1-Fc at the time of ischemia in non-reperfused myocardium lowers cardiomyocyte injury by 50 percent at 4 days post-MI, by reducing necrosis, apoptosis, and inflammatory cell infiltration [1]. In addition, acute treatment with EFNA1-Fc results in a 46 percent decrease in infarct size and improved cardiac function compared to untreated myocardium [3]. The underlying mechanism(s) by which EFNA1-Fc promotes tissue salvage, however, remain(s) unknown.

Previously, our group has successfully optimized and mapped both intact and enzymatically digested endogenous EFNA1 in cardiac tissue, pre- and post-MI, using MALDI mass spectrometry imaging (MSI) [4]. MALDI-MSI provides a means to characterize the spatial distribution of proteins in tissue sections, previously restricted to histology and immunohistochemistry. Immunohistochemical techniques are particularly limited in the number of proteins that can be identified in a single tissue section due to antibody specificity, subcellular compartmentalization, and color compatibility of the chromogen/fluorescent labels [5]. MALDI-MSI provides both qualitative and quantitative power of analysis, with the capability of detecting multiple metabolites and/or proteins in a single tissue section. A variety of applications using MALDI-MSI include targeting exogenous compounds in biological systems (i.e., cocaine, methadone, and naltrexone), growth hormones, and antibiotics (i.e., Rifampicin) [6,7,8,9,10]. Despite the extensive literature on the use of MALDI-MSI for the quantitation of small molecules, limited studies have been reported for the quantitation of proteins. Isotope labeling such as ^2^D, ^13^C, ^15^N, or ^18^O, the use of a chemical tag (i.e., p-hydroxymercuribenzoic acid) [11,12], or label-free methods [13] are viable MALDI-MSI methods for protein quantitation. For example, MALDI coupled with multiple reaction monitoring and liquid chromatography/mass spectrometry (LC/MS) has been used for imaging and quantification of myelin in rat brain tissue [14].

The study of the anatomical distribution of endogenous EFNA1 in cardiac tissue is of high clinical relevance to elucidate its role in the preservation of structural and functional cardiomyocyte integrity. We have previously optimized the conditions. Thus, the purpose of the present study was to optimize a robust MALDI-MSI method for the relative quantitation of endogenous EFNA1 levels in healthy murine cardiac muscle. The present method for relative quantitation will provide a tool to delve deeper into the role of EFNA1 in cardiac tissue, both in healthy and post-MI conditions, as well as upon administration of therapeutic agents.

## 2. Results

### 2.1. MALDI-MSI Optimization for the Identification of EFNA1 in Tissues

Enzymatic digestion has long been used in bottom-up proteomics, with trypsin being the gold-standard protease [15]. Four different experimental conditions of the EFNA1-His tag standard digestion were screened to determine the optimal conditions: (1) concentrations of the standard, (2) concentrations of trypsin, (3) duration of digestion, and (4) in-solution versus on-surface digestion. Optimization of trypsin digestion and MALDI matrix conditions were performed to ensure accurate and reproducible identification of EFNA1-His tag standard’s (*n* = 5) tryptic peptides. This allowed the identification of endogenous EFNA1 in cardiac tissue with high confidence in three independent samples. Table 1 lists a summary of the percent coverage results for EFNA1-His tag standards that were pipetted directly onto an ITO slide after a 4 h digestion of varying EFNA1 and trypsin concentrations. The peptides mixed with CHCA only provided a maximum of 9 percent coverage. Using the SA matrix with a trypsin concentration of 40 µg/mL and 100 µg/mL EFNA1 provided the greatest percent coverage, ranging from 19–49 percent after a 4 h digestion.

**Figure 1:**
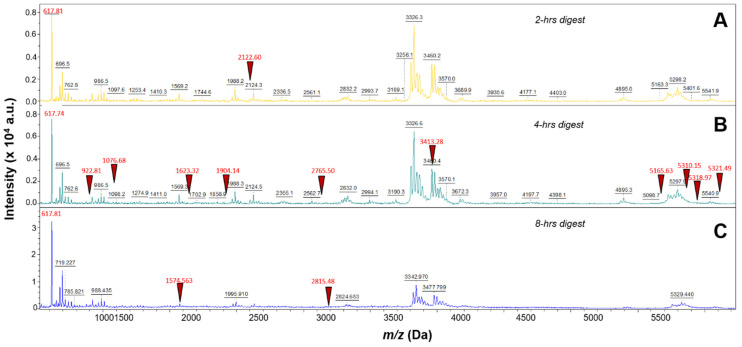
Mass spectra of EFNA1-His tag standard tryptic peptides obtained from three different digestion times. EFNA1-His tag standard at a concentration of 100 µg/mL was trypsin digested at 40 µg/mL for 2- (**A**), 4- (**B**), or 8- (**C**) hours. The red triangles represent some of the EFNA1 signature peptides.

Three different incubation time points were performed using 40 µg/mL trypsin digestion and 100 µg/mL EFNA1-His tag: 2 h, 4 h, and 8 h. As shown in Figure 1A,C, the 4 h digestion provided higher intensity peptide profiles compared to the 2 h and 8 h digestions. The percent coverage for the 2 h digestion was 15 percent and matched 3 EFNA1-His tag standard peptides (sequences 2, 6, and 26 in Supplementary Table S1), whereas the 4 h digestion provided 65 percent coverage, with 11 peptides matching EFNA1-His tag standard (sequences 2, 6, 9, 11, 20, 24, 37, 44, 65, 66, and 67 in Supplementary Table S1), and the 8 h digest exhibited 24 percent coverage with three peptides matching EFNA1-His tag standard (sequences 6, 16 and 38 in Supplementary Table S1).

**Figure 2:**
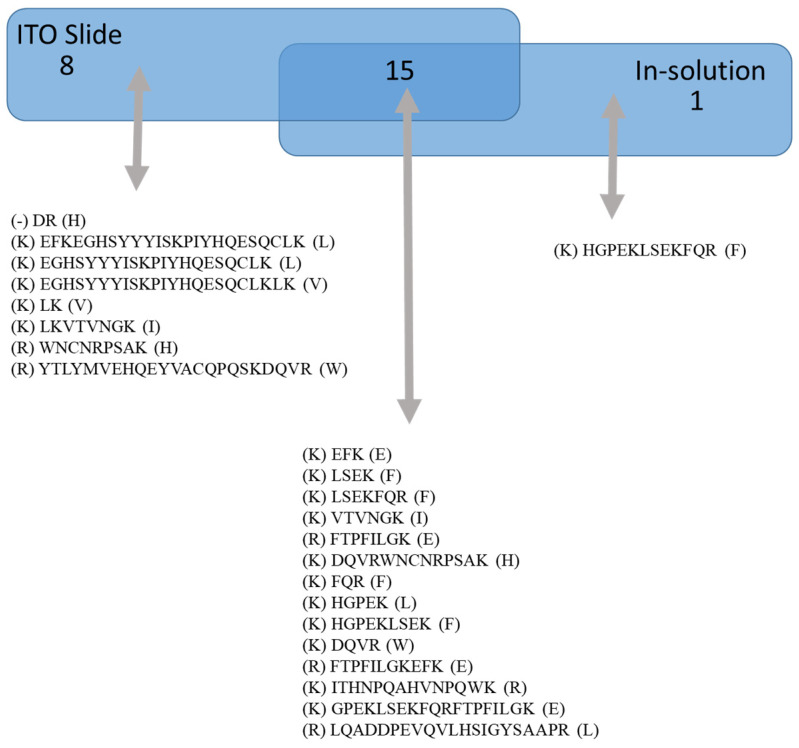
ITO slide vs. in solution digestion of EFNA1-His tag standard. The diagram shows the sequences of peptides identified by trypsin digestion of EFNA1-His tag standard on an ITO slide versus those detected with in-solution digestion.

**Figure 3:**
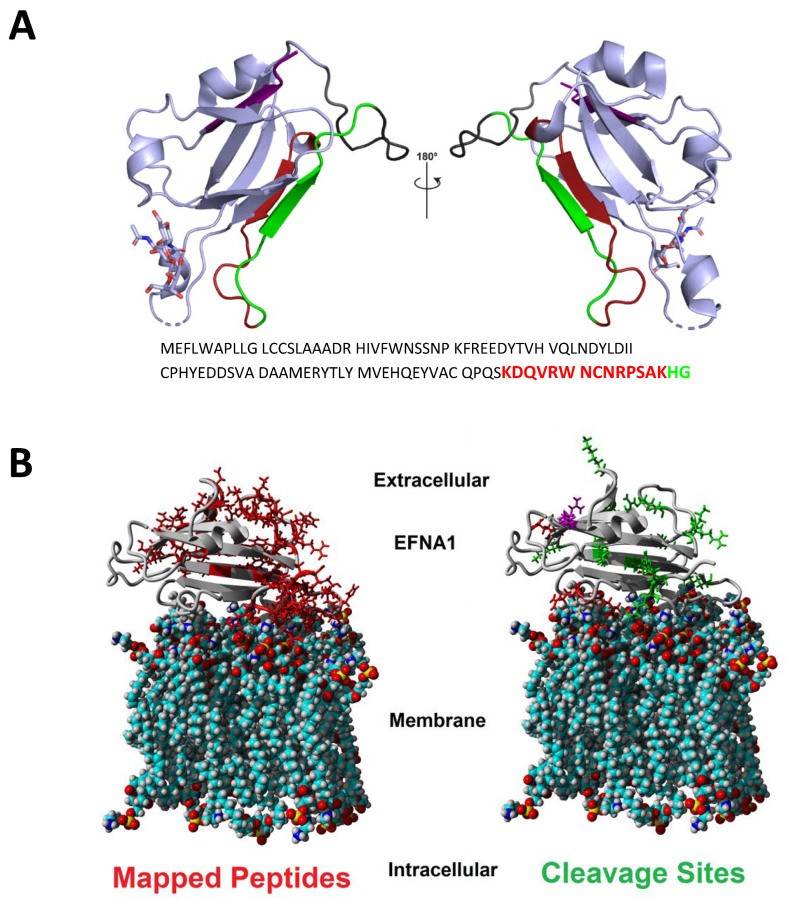
Ribbon (**A**) and calotte model (**B**) representations of EFNA1 and location of key peptide fragments. The peptide fragments identified using imaging in both cardiac tissue and EFNA1-His tag standards are highlighted in the structure with corresponding colors in the amino acid sequence (**A**). The calotte model shows the location of mapped peptides (left in red) and cleavage sites (right in green) relative to their position in the plasma membrane (**B**). EFNA1 models were generated using PyMOL version 1.8 and PDB 3CZU (https://www.rcsb.org/ (accessed on 15 August 2017)) [16].

A comparison between the EFNA1-His tag peptides for native (pipetted on a slide) versus in-solution (denaturing conditions) is shown in Figure 2. Peptides listed under “on ITO slide” include both cardiac tissue and EFNA1-His tag standards, whereas “in-solution digestion” only includes EFNA1-His tag standards. The results showed one unique peptide sequence detected with the in-solution digestion, while 15 overlapped between the two methods. Moreover, in the cardiac tissue samples and the endogenous standards on the ITO slide, there were eight unique peptides detected. To further understand differences in digestion peptide profiles, we proceeded to dimensionally locate each of the peptide fragments within the reported ribbon structure for EFNA1 (Figure 3A) as well as relative to the plasma membrane (Figure 3B).

### 2.2. EFNA1 Peptides Identified in Trypsin Digests of Cardiac Tissue

The ribbon model of EFNA1 (Figure 3A) depicts the specific sequences of the EFNA1 tryptic peptides detected in tissue samples and EFNA1-His tag standards, and the space-filling calotte model shows the location of the mapped peptides and cleavage sites relative to the plasma membrane (3B). The peptides identified were primarily located on the G-H loop, which is inherently flexible and thus freely accessible to the trypsin protease. These peptides were blasted for uniqueness and were found to be unique for EFNA1 with greater than 95% confidence. The 17 peptides identified in tissue and the EFNA1-His tag protein are depicted in Supplementary Table S1.

### 2.3. Relative Quantitation of EFNA1 Using MALDI-MSI

**Figure 4:**
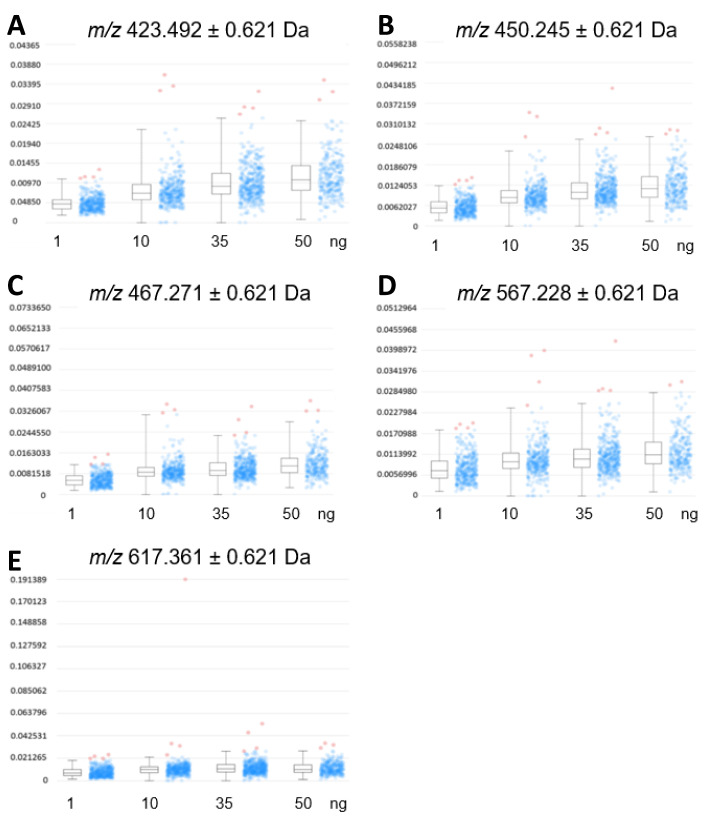
Expression profiles of EFNA1-His tag standard tryptic fragments. The intensity distributions of the peptides *m*/*z* 423.5 (**A**), 450.3 (**B**), 476.3 (**C**), 567.2 (**D**), and 617.4 Da (**E**) are shown at 1 ng, 10 ng, 35 ng, and 50 ng concentrations of the standard, respectively.

A relative comparison of the expression profiles in cardiac tissue (Figure 4) from three mice to the intensity profile distribution of EFNA1-His tag standards allowed for the relative quantification of endogenous ephrin A1 expression. Increasing EFNA1 concentrations corresponded to a concomitant increase of the mean intensity profiles, but the response was not linear and likely attributable to matrix selection [17,18,19].

As expected, MALDI-MSI consistently detected only 17 of the 75 possible ephrin A1 peptide fragments expected from the theoretical full digestion of the protein (Figure 3 and Table 2). These 17 consistently identified peptides were assigned as the signature fragments and utilized for relative quantitation.

**Figure 5:**
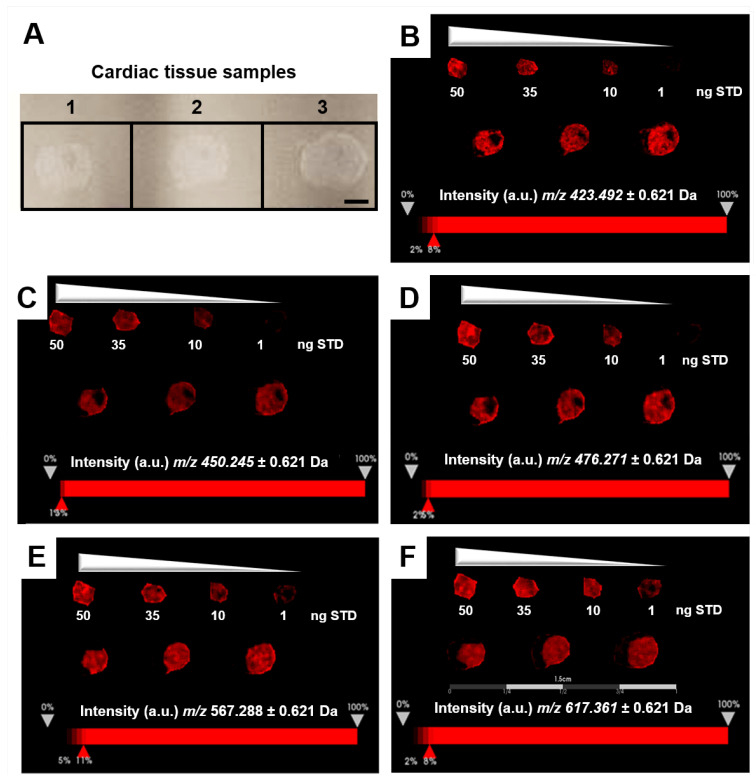
MALDI-MSI of EFNA1-tryptic peptides in the His tag standard and cardiac tissue samples on ITO slide. (**A**) Image of samples on ITO slide (scale bar = 2 mm). (**B**–**F**) Reconstructed chemical ion images of the EFNA1-tryptic peptides *m*/*z* 423.5 (**B**), 450.2 (**C**), 476.3 (**D**), 567.2 (**E**), and 617.4 (**F**) at 50, 35, 10 and 1 ng of the His tag standard (**top**) and three independent cardiac tissue samples (**bottom**) (scale bar 1.5 cm).

**Figure 6:**
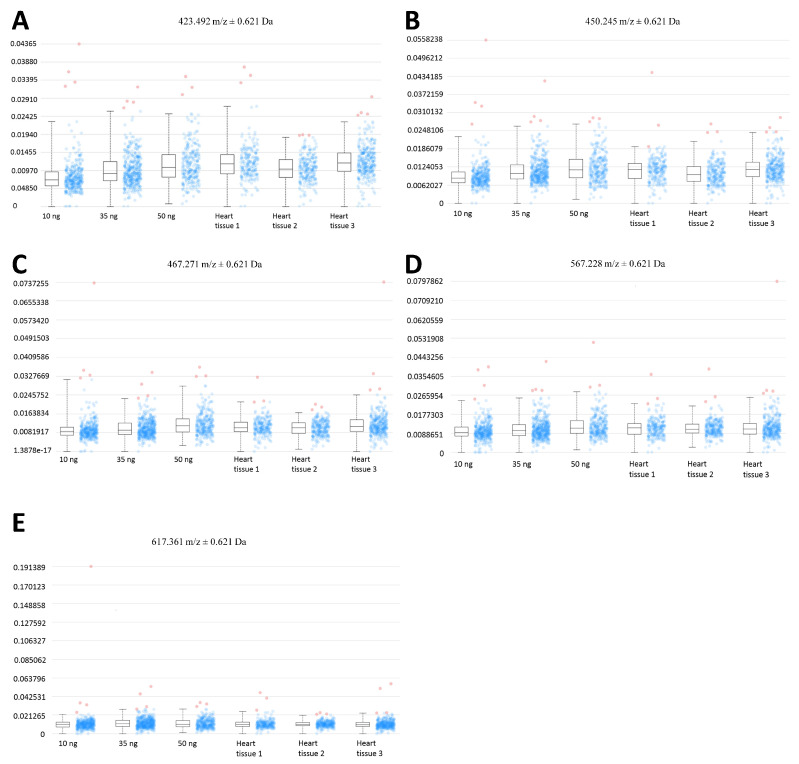
Expression profiles of EFNA1 tryptic peptide fragments in standards vs. cardiac tissue samples. The intensity distribution for the peptides *m*/*z* 423.5 (**A**), 450.3 (**B**), 476.3 (**C**), 567.2 (**D**), and 617.4 Da (**E**) are compared across different amounts of the analytical his tag standards (at 10 ng, 35 ng, and 50 ng), and in 3 independent samples of healthy mouse cardiac tissue.

**Figure 7:**
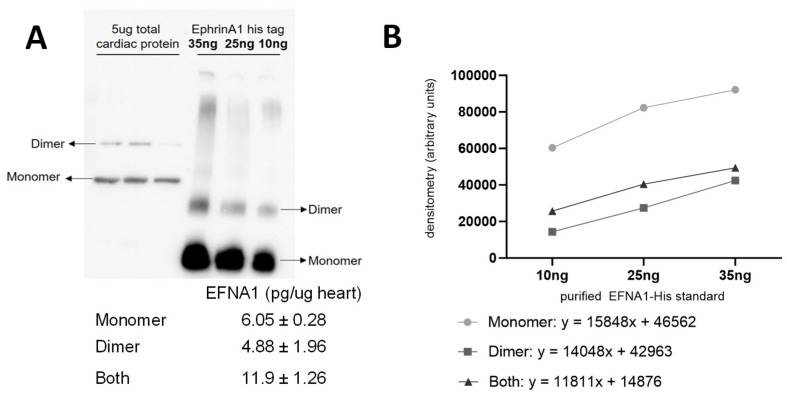
Representative Western blot of EFNA1 in mouse cardiac tissue relative to purified EFNA1-His standard (**A**) and EFNA1-FLAG and EFNA1-CC (coiled-coil) constructs made in *E. coli* (**B**). The image shows the relative amounts of EFNA1 monomer and dimer protein in 5 µg of heart tissue (left in triplicate) compared to 100, 25, and 25 ng of EFNA1-His standard (right in descending order left to right) as well as EFNA1-FLAG and EFNA1-CC.

The relative quantitation of all EFNA1 peptides in three healthy hearts is summarized in Figure 5. Cardiac tissue mean expression levels were compared to those expression levels shown in the EFNA1-His tag standard at concentrations 1, 10, 35, and 50 ng. From the reconstructed chemical ion images of the tryptic peptides identified in both endogenous EFNA1 and the EFNA1-His tag standard (Figure 6), we estimated the endogenous levels of EFNA1 to be approximately 50 ng per section in healthy murine cardiac tissue from three mice, which equates to 12 pg/µg of homogenized tissue. Figure 7A is a representative western blot showing the predominance of the monomeric form in the purified recombinant EFNA1-His compared to the whole mouse heart homogenate. Figure 7B shows the densitometry-derived equations used to extrapolate the relative quantity of EFNA1 in mouse heart tissue sections in Figure 7A.

## 3. Discussion

The purpose of this study was to perform relative quantitation of all endogenous EFNA1 proteoforms combined using MALDI-MSI. Comparing our data with western blotting, a routinely used low-cost method, indicates that there is approximately 50 ng of the protein per cardiac tissue section of 9.43 mm^3^ per volume. An alternative, routinely used semi-quantitative western blotting approach to calculate this validates this assessment, showing that there is approximately 60 pg/5 µg heart tissue. Using MALDI-MSI as a relative quantitation tool with protein expression levels for peptides of interest in tissue yields previously unachievable data, although with some limitations. Optimization of the methodology, specifically matrix selection and the trypsin digestion protocol, plays a significant role in the ability to obtain accurate, reliable protein quantitation data from biological systems. Specifically, the 4 h digestion using SA matrix and 40 µg/mL of trypsin solution yielded the maximum number of missed cleavages—three. During the process of matrix optimization, we observed the formation of a polymerization interference, possibly due to polymer contaminant, in the CHCA samples. Importantly, the effect was not seen when using the SA matrix (Supplementary Table S1), further supporting the use of SA as the most efficient matrix for studying both EFNA1-His tag standard and endogenous EFNA1 at a trypsin concentration of 40 µg/mL.

A comparison of the sequences of peptides identified by trypsin digestion of EFNA1-His tag standard on an ITO slide versus those detected with in-solution digestion showed that there was only one unique peptide sequence detected with the in-solution digestion, while there were 15 identified as overlapping between the two methods. There were eight unique peptides in cardiac tissue samples and the endogenous standards on the ITO slide. The dimensional location of each peptide fragment within the reported ribbon structure for EFNA1 (Figure 3A), as well as relative to the plasma membrane (Figure 3B), sheds light on the differential digestion profiles. In particular, it is interesting to note that the peptides were located primarily on the G-H loop, which is inherently flexible and thus openly accessible to the trypsin protease. A BLAST search of the 17 peptides (Supplementary Table S1) identified in tissue and the EFNA1-His tag showed them to be unique for EFNA1 with greater than 95% confidence.

Relative comparison of the tissue expression profiles to the intensity profile distribution of EFNA1-His tag standards allowed for the relative quantification of endogenous ephrin A1 expression in a mouse heart tissue section (Figure 4). Increasing EFNA1 concentrations corresponded to an increased mean intensity profile, but the response was not linear and likely attributable to matrix selection [17,18,19]. Matrix-driven ion suppression presents a significant challenge in MALDI/MSI of biological samples due to chemical interferences from both the matrix per se and the complexity of components within the sample [20]. Although necessary for relative quantification purposes, complete trypsin digestion of native proteins in tissue samples is difficult compared to digestions of denatured proteins, as performing native protein digestion often results in numerous missed cleavage sites. This is due to secondary and tertiary structural elements of the target protein proteoforms that limit trypsin’s accessibility to Arg and Lys residues [16].

MALDI-MSI-derived relative quantitation of all EFNA1 peptides is summarized in Figure 5. As previously described, the cardiac tissue mean expression levels were compared to those expression levels shown in the EFNA1-His tag standard. From the reconstructed chemical ion images of the tryptic peptides identified in both endogenous EFNA1 and the EFNA1-His tag standard (Figure 6), we estimated the endogenous levels of EFNA1 to be approximately 50 ng per section in healthy murine cardiac tissue, which equates to 12 pg/µg of homogenized tissue. Using western blotting (Figure 7), we were able to show the predominance of the monomeric form in the purified recombinant EFNA1-His compared to the whole mouse heart homogenate and that the densitometry-derived equations could be used to semi-quantitatively extrapolate the relative quantity of endogenous EFNA1 in mouse heart tissue sections.

The ability to measure the relative quantitative expression profiles of proteins while maintaining their spatial integrity allows us to assess protein tissue distribution profiling upon physiological perturbations, tissue injury, or disease. Future work in our group will expand on measuring the expression profile of EFNA1 interactions with other target proteins, such as with the associated EphA family of receptors before and after MI. Additional studies in progress continue to explore the potential for EFNA1-Fc to be used as a novel therapeutic for the treatment of MI.

## 4. Materials and Methods

### 4.1. Reagents

Optima grade water, acetonitrile, and trifluoroacetic acid were purchased from Fisher Scientific (Hampton, NH, USA). 3,5-dimethoxy 4-hydroxycinnamic acid (sinapinic acid, SA BCBR3510V 85429) and α-cyano-4-hydroxycinnamic acid (CHCA BCBK18424 70990) were purchased from Sigma-Aldrich (St Louis, MO, USA). Indium tin oxide (ITO)–coated conductive glass microscope slides were purchased from Bruker Daltonics (Billerica, MA, USA). Sequencing grade trypsin (V511A) was purchased from Promega (Madison, WI, USA). Accu-Edge^®^ low profile blades (4689) were purchased from VWR (Radnor, PA, USA). EFNA1-His tag standard was purchased from Sino Biological (50593-M08H, Beijing, China). Sodium bicarbonate was purchased from Sigma-Aldrich (SLB53027V S6014, St Louis, MO, USA). Ethanol was purchased from Acros Organics (B0534756 64-17-5, Geel, Belgium). Bio-Rad Bradford protein assay kit (Product #1856210, Hercules, CA, USA) and Bio-Rad Immuno-blot polyvinylidene difluoride (PVDF) membranes were acquired from Fisher (Product #PV4HY00010, Hampton, NH, USA). Santa Cruz Biotechnology (Product number sc-377362, Dallas, TX, USA) primary antibody anti-EFNA1 (SC-911) used for western blots. Secondary antibody anti-rabbit IgG was purchased from R&D Systems (Product # HAF008, Minneapolis, MN, USA). SuperSignal West Femto Maximum Sensitivity Substrate (Thermo Scientific, Product #34095, Waltham, MA, USA). Protein extraction reagent type 4 was used for protein extraction [21] (Sigma-Aldrich # C0356-4BTL, St Louis, MO, USA).

### 4.2. Animal Use, Surgical Procedure, and Tissue Collection

Experimental research protocols were approved by the East Carolina University Institutional Animal Care and Use Committee (IACUC), following the guidelines of the National Institutes of Health for the Care and Use of Laboratory Animals. Animal care was maintained by the Department of Comparative Medicine at The Brody School of Medicine, East Carolina University. Six male and four female B6129SF2/J (stock #101045) 8–12-week-old male mice were purchased from the Jackson Laboratories (Bar Harbor, MN, USA). Mice were housed with 12 h/12 h light/dark cycle conditions and were fed ad libitum. Animals were anesthetized with pentobarbital (100 mg/Kg BW) and sacrificed for tissue harvest. Whole hearts were thoroughly perfused with ice-cold PBS (10–15 mL, for 2–3 min), wrapped loosely in foil, and snap-frozen in liquid nitrogen. Samples were then stored at –80 °C prior to analysis.

### 4.3. Cryosectioning

Frozen tissues were mounted on a cryostat chuck with water and allowed to freeze at −80 °C for 5 min. Sections that were 10 µm thick and resulted in an average volume of 9.425 mm^3^ were then thaw-mounted on an Indium-Tin-Oxide (ITO) slide. Slides were washed for 30 s in 70 percent, 90 percent, and 95 percent ethanol and then placed in a vacuum desiccator with Drierite for 30 min [22]. EFNA1-His tag standards were added to the slide for relative quantitation.

### 4.4. Analytical Standards

EFNA1-His tag standards were diluted in water prior to use. Concentrations of 10, 25, 50, and 100 µg/mL EFNA1-His tag standards were used for optimization (matrix selection and tryptic digestion time). For relative quantitation of endogenous EFNA1 in tissue, the EFNA1-His tag standard was diluted at concentrations of 0.001, 0.1, 1, 10, 35, 50, and 100 µg/mL. In all cases, 1 µL of the standard was pipetted directly onto ITO slides and allowed to dry in an incubator at 37 °C. Slides were incubated for a duration of 1, 2, 4, 6, and 8 h. At each time point, four different trypsin concentrations (10, 20, 40, or 100 µg/mL) were utilized to determine the optimal digestion concentration for EFNA1.

### 4.5. Trypsin

A stock solution containing 20 µg of trypsin was resuspended in 20 µL of 50 mM acetic acid, and then 333 µL of ammonium bicarbonate containing 0.01 percent ammonium hydroxide, 40 µL of acetonitrile, and 67 µL of 100 mM acetic acid. This was applied to the tissue on the slides, which were incubated at 30 °C for 30 min. 

### 4.6. In-Solution Protein Digestion

In-solution trypsin digestions were performed using the UAB mass spectrometry/proteomics method, ref. [22] available at: https://www.uab.edu/medicine/msproteomics/images/eduProtPDF/cellstrypsin.pdf (accessed on 2 October 2017). Briefly, non-denatured samples were exposed to 200 mM DTT/50 mM Tris-HCl followed by 200 mM iodoacetamide/50 mM Tris-HCl in the dark. DTT/Tris-HCl was added one more time to consume any unreacted iodoacetamide. The same trypsin solution used for EFNA1-His tag standards and tissue was added at a ratio of 1:50 (trypsin/protein) and incubated at 37 °C for 18 h. The same experimental procedure was followed for denatured samples, but with boiling for 5 min at 95 °C before the DTT/Tris-HCl incubation.

### 4.7. Tissue Trypsin Digestion

Tryptic digestion of tissues was performed with the same trypsin solution, activation time, and temperature as used for the digestion of the analytical standard spiked onto the slide in order to control for effects on ion suppression. The HTX TM sprayer (HTX Technologies LLC, Chapel Hill, NC, USA) was used for the application of trypsin to the tissue on the ITO slides with a syringe pump. Parameters were set as follows: a flow rate of 60 µL/min for two passes at 600 mm/min, track spacing of 2 mm with a crisscross pattern at 12 psi, a nozzle height of 40 mm, and a 3 L/min gas flow rate.

### 4.8. In-Gel Protein Digestion

Recombinant EFNA1-His and mouse heart samples were run on SDS as well as native gels. The gels were stained, and appropriate bands were excised and transferred to individual 2 mL microcentrifuge tubes. After washing the gel pieces, they were destained using 100 mM (NH_4_)HCO_3_/50% acetonitrile. The pieces were then treated with 100% acetonitrile until they shrank and became white. Gel pieces were then treated with 5 mM DTT to reduce the disulfides and then alkylated with 50 mM IAA. Trypsin was used to digest the gel pieces. 0.02 µg/µL of trypsin was added to each microcentrifuge tube and incubated at 37 °C for 30 min.

### 4.9. Matrix Application

Our previous study determined that sinapinic acid (SA) is a more effective matrix than α-cyano-4-hydroxycinnamic acid (CHCA) for the identification of intact EFNA1 [4]. To optimize matrix selection for the trypsinized tissues, either CHCA or SA was pipetted (1 µL) on top of the analytical standard and allowed to dry at room temperature. SA was selected for all subsequent imaging experiments, in-solution experiments, and relative quantitation. The matrix was added to ITO slides using the HTX TM sprayer, with parameters set to 30 °C for 12 passes and a flow rate of 0.1 mL/min at 750 mm/min. Track spacing was set to 2 mm using a crisscross pattern delivered at 10 psi with a gas flow rate of 0.1 mL/min and nozzle height of 40 mm.

### 4.10. MALDI Settings

Prior to trypsin digestion, EFNA1-His tag standards at concentrations of 0.001, 0.1, 1, 10, 35, 50, and 100 µg/mL were pipetted onto the slide and allowed to dry. Dry slides were scanned using a Bruker MALDI-TOF/TOF Autoflex Speed (Bruker, Billerica, MA, USA) at 2400 dpi with teach markings at the corners to denote orientation and then loaded into FlexImaging 5.0 for the region of interest selection. All experiments were completed using reflectron positive mode with laser power optimized for signal intensity (10^4^) and a mass range of 0 to 5000. During method optimization, 5000 spectra were collected per sample at a frequency of 2000 Hz. For in-solution digestion experiments, 8000 spectra were collected for each sample, and for relative quantitation of endogenous EFNA1, 8000 spectra were collected every 75 µm. FlexImaging 5.0 was used to collect the data along with FlexControl 3.4 and FlexAnalysis 4.0 for EFNA1 spectra.

### 4.11. Data Analysis

The sequence of EFNA1-His tag was theoretically digested using the protein prospector database (http://prospector.ucsf.edu/ (accessed on 4 Novermber 2016)) based on the amino acid sequence and predicted cleavage sites. Parameters included digestion by trypsin with a max of 5 possible missed cleavages, no variable modifications, a peptide mass range of 400–5000 *m*/*z*, a minimum peptide length of 5, and MALDI-TOF/TOF instrumentation to provide *m*/*z* values and predicted sequences from EFNA1 tag standards. Protein prospector theoretical *m*/*z* data were compiled and compared to the experimental *m*/*z* values found. Spectra generated to determine optimal matrix and digestion time were analyzed with flexanalysis, calibrated using PEG 600 and angiotensin II, and normalized to the total ion current. Spectra were processed using a centroid algorithm with a signal-to-noise ratio of 3. Peak width was set at 2 *m*/*z* and height at 10 percent. All spectra were smoothed once with single baseline subtraction using the TopHat version 2.1.1 algorithm and were smoothed once using SavitzkyGolay set to a width of 0.2 *m*/*z* at one cycle.

### 4.12. Ribbon Model of EFNA1 and Image Analysis

The EFNA1 ribbon model was generated based on the PDB file 3CZU [16]. Mass lists were generated using SCiLS for healthy control cardiac tissue and EFNA1-His tag standards. Spectra in SCiLS Lab were aligned to the mean spectra, then exported as overview spectra in a CSV file with an intensity threshold of 6. EFNA1 tryptic peptides were identified using protein prospector MS-Bridge in the EFNA1-His tag standard, cardiac tissue, and in-solution mass lists. Relative quantitation of EFNA1 was conducted using SCiLS Lab software version 6.01.10183, and known *m*/*z* from tryptic peptides from EFNA1 were selected. Intensity box plots and reconstructed ion images were generated for each tryptic peptide from EFNA1-His tag standard and endogenous EFNA1.

## Figures and Tables

**Table 1:** Summary of percent coverage for EFNA1-His tag standards comparing CHCA and SA matrices in two optimization protocols: 100 µg/mL EFNA1-His tag with varying trypsin concentrations (**top**) and varying EFNA1-His tag concentrations using 40 µg/mL trypsin (**bottom**).

Matrix	EphrinA1 Concentration	Trypsin Concentration	Coverage Percentage	Sequence Identified (See Supplemental Table S1)
CHCA	100 µg/mL	20 µg/mL	0–9%	5, 8
CHCA	100 µg/mL	40 µg/mL	0–3%	4, 8, 18
CHCA	100 µg/mL	100 µg/mL	0–1%	4, 8, 27
SA	100 µg/mL	20 µg/mL	25–29%	14, 43, 45, 48, 51
SA	100 µg/mL	40 µg/mL	19–49%	4, 7, 11, 13, 16, 22, 26, 29, 32, 39, 43, 44, 45, 46, 47, 48, 51, 52, 57, 69, 70, 71, 72
SA	100 µg/mL	100 µg/mL	0–34%	7, 18
CHCA	10 µg/mL	40 µg/mL	2–3%	3, 5, 10
CHCA	25 µg/mL	40 µg/mL	11–19%	4, 5, 6, 10, 19
CHCA	50 µg/mL	40 µg/mL	0–3%	4, 10
CHCA	100 µg/mL	40 µg/mL	0–9%	4, 8, 18
SA	10 µg/mL	40 µg/mL	17–57%	7, 8, 12, 15, 27, 42, 45, 53, 71, 75
SA	25 µg/mL	40 µg/mL	5%	17
SA	50 µg/mL	40 µg/mL	10–33%	7, 18, 61
SA	100 µg/mL	40 µg/mL	19–49%	4, 7, 11, 13, 16, 22, 26, 29, 32, 39, 43, 44, 45, 46, 47, 48, 51, 52, 57, 69, 70, 71, 72

**Table 2:** List of tryptic peptides detected in both cardiac tissue samples and EFNA1-His tag standard.

Start	End	Observed	Mr(expt)	Mr(calc)	Delta	M	Peptide Detected
119	121	423.492	422.4846	422.4755	0.009	0	(K) DQVR (W)
108	110	450.521	449.5136	449.5041	0.01	0	(K) DQVRWNCNRPSAK (H)
104	107	476.553	475.5456	475.5365	0.009	0	(K) EFK (E)
86	89	517.566	516.5586	516.5487	0.01	0	(K) FQR (F)
99	103	567.626	566.6186	566.6074	0.011	0	(K) HGPEK (L)
145	150	617.727	616.7196	616.7076	0.012	0	(K) HGPEKLSEK (F)
143	150	859.063	858.0556	858.0375	0.018	1	(K) HGPEKLSEKFQR (F)
104	110	908.051	907.0436	907.0253	0.018	1	(K) HGPEKLSEKFQRFTPFILGK (E)
111	118	923.15	922.1426	922.121	0.022	0	(K) ITHNPQAHVNPQWK (R)
99	107	1025.153	1024.146	1024.129	0.017	1	(K) LSEK (F)
90	98	1076.229	1075.222	1075.202	0.02	0	(K) LKVTVNGK (I)
111	121	1327.619	1326.612	1326.581	0.03	1	(K) LSEKFQR (F)
99	110	1456.654	1455.647	1455.617	0.029	2	(K) VTVNGK (I)
86	98	1574.772	1573.765	1573.735	0.03	1	(R) FTPFILGK (E)
151	164	1613.784	1612.777	1612.745	0.032	0	(R) FTPFILGKEFK (E)
166	186	2267.519	2266.512	2266.466	0.046	0	(R) LQADDPEVQVLHSIGYSAAPR (L)
99	118	2360.782	2359.775	2359.723	0.052	3	(R) WNCNRPSAK (H)

## Data Availability

All data supporting the findings of this study are included in this manuscript and Supplement Materials.

## Supplementary Materials

The supporting information can be downloaded at: https://www.mdpi.com/article/10.3390/ijms26041398/s1.

## References

[1] Pasquale E.B. (2008). Eph-Ephrin Bidirectional Signaling in Physiology and Disease. Cell.

[2] Dries J.L., Kent S.D., Virag J.A.I. (2011). Intramyocardial administration of chimeric ephrinA1-Fc promotes tissue salvage following myocardial infarction in mice. J. Physiol..

[3] DuSablon A., Parks J., Whitehurst K., Estes H., Chase R., Vlahos E., Sharma U., Wert D., Virag J. (2017). EphrinA1-Fc attenuates myocardial ischemia/reperfusion injury in mice. PLoS ONE.

[4] Lefcoski S., Kew K., Reece S., Torres M.J., Parks J., Reece S., Brás L.E.d.C., Virag J.A.I. (2018). Anatomical-Molecular Distribution of EphrinA1 in Infarcted Mouse Heart Using MALDI Mass Spectrometry Imaging. J. Am. Soc. Mass Spectrom..

[5] Cornett D.S., Reyzer M.L., Chaurand P., Caprioli R.M. (2007). MALDI imaging mass spectrometry: Molecular snapshots of biochemical systems. Nat. Methods.

[6] Prentice B.M., Chumbley C.W., Caprioli R.M., Kataoka K., Andrén P.E., Hummon A.B., Takats Z., Lee C.Y., Kelleher N.L., Heeren R.M.A. (2016). Absolute Quantification of Rifampicin by MALDI Imaging Mass Spectrometry Using Multiple TOF/TOF Events in a Single Laser Shot. J. Am. Soc. Mass Spectrom..

[7] Chumbley C.W., Reyzer M.L., Allen J.L., Marriner G.A., Via L.E., Barry C.E., Caprioli R.M. (2016). Absolute Quantitative MALDI Imaging Mass Spectrometry: A Case of Rifampicin in Liver Tissues. Anal. Chem..

[8] Reich R.F., Cudzilo K., Levisky J.A., Yost R.A. (2010). Quantitative MALDI-MS*^n^*analysis of cocaine in the autopsied brain of a human cocaine user employing a wide isolation window and internal standards. J. Am. Soc. Mass Spectrom..

[9] Bucknall M., Fung K.Y.C., Duncan M.W. (2002). Practical quantitative biomedical applications of MALDI-TOF mass spectrometry. J. Am. Soc. Mass Spectrom..

[10] Teklezgi B.G., Pamreddy A., Baijnath S., Kruger H.G., Naicker T., Gopal N.D., Govender T. (2019). Time-dependent regional brain distribution of methadone and naltrexone in the treatment of opioid addiction. Addict. Biol..

[11] Bantscheff M., Dümpelfeld B., Kuster B. (2004). Femtomol sensitivity post-digest18O labeling for relative quantification of differential protein complex composition. Rapid Commun. Mass Spectrom..

[12] Kutscher D.J., Bettmer J. (2009). Absolute and Relative Protein Quantification with the Use of Isotopically Labeled *p*-Hydroxymercuribenzoic Acid and Complementary MALDI-MS and ICPMS Detection. Anal. Chem..

[13] Albalat A., Stalmach A., Bitsika V., Siwy J., Schanstra J.P., Petropoulos A.D., Vlahou A., Jankowski J., Persson F., Rossing P. (2013). Improving peptide relative quantification in MALDI-TOF MS for biomarker assessment. Proteomics.

[14] Nicklay J.J., Harris G.A., Schey K.L., Caprioli R.M. (2013). MALDI Imaging and in Situ Identification of Integral Membrane Proteins from Rat Brain Tissue Sections. Anal. Chem..

[15] Zhang Y., Fonslow B.R., Shan B., Baek M.-C., Yates J.R. (2013). Protein Analysis by Shotgun/Bottom-up Proteomics. Chem. Rev..

[16] Himanen J.P., Yermekbayeva L., Janes P.W., Walker J.R., Xu K., Atapattu L., Rajashankar K.R., Mensinga A., Lackmann M., Nikolov D.B. (2010). Architecture of Eph receptor clusters. Proc. Natl. Acad. Sci. USA.

[17] Yang J., Caprioli R.M. (2011). Matrix Sublimation/Recrystallization for Imaging Proteins by Mass Spectrometry at High Spatial Resolution. Anal. Chem..

[18] Parker L., Engel-Hall A., Drew K., Steinhardt G., Jr D.L.H., Jabon D., McMurry T., Angulo D.S., Kron S.J. (2007). Investigating quantitation of phosphorylation using MALDI-TOF mass spectrometry. J. Mass Spectrom..

[19] Knochenmuss R., Karbach V., Wiesli U., Breuker K., Zenobi R. (1998). The matrix suppression effect in matrix-assisted laser de-sorption/ionization: Application to negative ions and further characteristics. Rapid Commun. Mass Spectrom..

[20] Kukret H., Amuthavalli K. (2020). MicroRNA-34a causes ceramide accumulation and effects insulin signaling pathway by targeting ceramide kinase (CERK) in aging skeletal muscle. J. Cell Biochem..

[21] Brás L.E.d.C., Ramirez T.A., DeLeon-Pennell K.Y., Chiao Y.A., Ma Y., Dai Q., Halade G.V., Hakala K., Weintraub S.T., Lindsey M.L. (2013). Texas 3-Step decellularization protocol: Looking at the cardiac extracellular matrix. J. Proteom..

[22] Mobley J. (2008). General Trypsin Digestion Using Urea Protocol for Cell Lysates; LCMS on LTQ. https://www.uab.edu/medicine/msproteomics/images/eduProtPDF/cellstrypsin.pdf.

