# Supplementary material for: Relative Quantitation of EFNA1 Expression in Mouse Heart Tissue Histologic Sections Using MALDI-MSI
Source: Int J Mol Sci. 2025 Feb 7;26(4):1398. doi: 10.3390/ijms26041398 (PMC11855005; doi:10.3390/ijms26041398)
Supplement: Supplementary file 1 [file ijms-26-01398-s001.zip › ijms-3398880-supplementary.pdf]

Supplement
Table S1

Sequence Number	Sequence	Matrix
1	(-)DR(H)	
2	(K)LV(V)	
3	(K)EFK(E)	CHCA
4	(K)FQR(F)	CHCA/ SA
5	(K)LSEK(F)	CHCA
6	(K)DQVR(W)	CHCA
7	(K)HGPEK(L)	SA
8	(K)VTVNGK(I)	CHCA/ SA
9	(K)LKVTVNGK(I)	
10	(K)LSEKFQR(F)	CHCA
11	(R)FTPFILGK(E)	SA
12	(K)HGPEKLSEK(F)	SA
13	(R)WNCNRPSAK(H)	SA
14	(R)FTPFILGKEFK(E)	SA
15	(R)HIVFWNSSNPK(F)	SA
16	(K)FQRFTPFILGK(E)	SA
17	(K)HGPEKLSEKFQR(F)	SA
18	(K)DQVRWNCNRPSAK(H)	CHCA/ SA
19	(-)DRHIVFWNSSNPK(F)	CHCA
20	(K)ITHNPQAHVNPQEK(R)	
21	(R)WNCNRPSAKHGPEK(L)	
22	(R)HIVFWNSSNPKFR(E)	SA
23	(K)FQRFTPFILGKEFK(E)	
24	(K)ITHNPQAHVNPQEKR(L)	
25	(K)LSEKFQRFTPFILGK(E)	
26	(-)DRHIVFWNSSNPKFR(E)	SA
27	(R)WNCNRPSAKHGPEKLSEK(F)	CHCA/ SA
28	(K)DQVRWNCNRPSAKHGPEK(L)	
29	(K)VTVNGKITHNPQAHVNPQEK(R)	SA
30	(K)LSEKFQRFTPFILGKEFK(E)	
31	(R)YTLYMVEHQEYVACQPQSK(D)	
32	(K)HGPEKLSEKFQRFTPFILGK(E)	SA
33	(K)VTVNGKITHNPQAHVNPQEKR(L)	
34	(K)LKVTVNGKITHNPQAHVNPQEK(R)	
35	(R)WNCNRPSAKHGPEKLSEKFQR(F)	
36	(K)EGHSYYYISKPIYHQESQCLK(L)	
37	(K)DQVRWNCNRPSAKHGPEKLSEK(F)	
38	(K)LKVTVNGKITHNPQAHVNPQEKR(L)	
39	(K)HGPEKLSEKFQRFTPFILGKEFK(E)	SA
40	(K)EGHSYYYISKPIYHQESQCLKLK(V)	
41	(R)YTLYMVEHQEYVACQPQSKDQVR(W)	
42	(K)EFKEGHSYYYISKPIYHQESQCLK(L)	SA

43	(K)DQVRWNCNRPSAKHGPEKLSEKFQR(F)	SA
44	(K)EFKEGHSYYYISKPIYHQESQCLKLK(V)	SA
45	(R)LQADDPEVQVLHSIGYSAHHHHHHHHHH(-)	SA
46	(K)EGHSYYYISKPIYHQESQCLKLKVTVNGK(I)	
47	(R)WNCNRPSAKHGPEKLSEKFQRFTPFILGK(E)	SA
48	(K)RLQADDPEVQVLHSIGYSAHHHHHHHHHH(-)	SA
49	(K)EFKEGHSYYYISKPIYHQESQCLKLKVTVNGK(I)	
50	(R)WNCNRPSAKHGPEKLSEKFQRFTPFILGKEFK(E)	
51	(R)EEDYTVHVQLNDYLDIICPHYEDDSVADAAMER(Y)	SA
52	(R)YTLYMVEHQEYVACQPQSKDQVRWNCNRPSAK(H)	SA
53	(R)FTPFILGKEFKEGHSYYYISKPIYHQESQCLK(L)	SA
54	(K)DQVRWNCNRPSAKHGPEKLSEKFQRFTPFILGK(E)	
55	(R)FTPFILGKEFKEGHSYYYISKPIYHQESQCLKLK(V)	
56	(K)FREEDYTVHVQLNDYLDIICPHYEDDSVADAAMER(Y)	
57	(K)FQRFTPFILGKEFKEGHSYYYISKPIYHQESQCLK(L)	SA
58	(R)YTLYMVEHQEYVACQPQSKDQVRWNCNRPSAKHGPEK(L)	
59	(K)FQRFTPFILGKEFKEGHSYYYISKPIYHQESQCLKLK(V)	
60	(R)FTPFILGKEFKEGHSYYYISKPIYHQESQCLKLKVTVNGK(I)	
61	(K)LSEKFQRFTPFILGKEFKEGHSYYYISKPIYHQESQCLK(L)	SA
62	(R)YTLYMVEHQEYVACQPQSKDQVRWNCNRPSAKHGPEKLSEK(F)	
63	(K)EGHSYYYISKPIYHQESQCLKLKVTVNGKITHNPQAHVNPQEK(R)	
64	(K)LSEKFQRFTPFILGKEFKEGHSYYYISKPIYHQESQCLKLK(V)	
65	(K)ITHNPQAHVNPQEKRLQADDPEVQVLHSIGYSAHHHHHHHHHH(-)	
66	(K)FQRFTPFILGKEFKEGHSYYYISKPIYHQESQCLKLKVTVNGK(I)	
67	(K)EGHSYYYISKPIYHQESQCLKLKVTVNGKITHNPQAHVNPQEKR(L)	
68	(R)YTLYMVEHQEYVACQPQSKDQVRWNCNRPSAKHGPEKLSEKFQR(F)	
69	(K)HGPEKLSEKFQRFTPFILGKEFKEGHSYYYISKPIYHQESQCLK(L)	SA
70	(K)EFKEGHSYYYISKPIYHQESQCLKLKVTVNGKITHNPQAHVNPQEK(R)	SA
71	(R)HIVFWNSSNPKFREEDYTVHVQLNDYLDIICPHYEDDSVADAAMER(Y)	SA
72	(K)EFKEGHSYYYISKPIYHQESQCLKLKVTVNGKITHNPQAHVNPQEKR(L)	SA
73	(K)VTVNGKITHNPQAHVNPQEKRLQADDPEVQVLHSIGYSAHHHHHHHHHH(-)	
74	(-)DRHIVFWNSSNPKFREEDYTVHVQLNDYLDIICPHYEDDSVADAAMER(Y)	
75	(K)LKVTVNGKITHNPQAHVNPQEKRLQADDPEVQVLHSIGYSAHHHHHHHHHH(-)	SA
